# Narrative language competence in children and adolescents with Down syndrome

**DOI:** 10.3389/fnbeh.2015.00283

**Published:** 2015-10-30

**Authors:** Marie Moore Channell, Andrea S. McDuffie, Lauren M. Bullard, Leonard Abbeduto

**Affiliations:** ^1^MIND Institute, University of California, DavisDavis, CA, USA; ^2^Department of Speech and Hearing Science, University of Illinois at Urbana–ChampaignChampaign, IL, USA

**Keywords:** Down syndrome, intellectual disability, language development, narrative language, neurodevelopmental disorders

## Abstract

This study was designed to examine the narrative language abilities of children and adolescents with Down syndrome (DS) in comparison to same-age peers with fragile X syndrome (FXS) and younger typically developing (TD) children matched by nonverbal cognitive ability levels. Participants produced narrative retells from a wordless picture book. Narratives were analyzed at the macrostructural (i.e., their internal episodic structure) and the microstructural (i.e., rate of use of specific word categories) levels. Mean length of utterance (MLU), a microstructural metric of syntactic complexity, was used as a control variable. Participants with DS produced fewer episodic elements in their narratives (i.e., their narratives were less fully realized) than the TD participants, although MLU differences accounted for the macrostructural differences between participant groups. At the microstructural level, participants with DS displayed a lower rate of verb use than the groups with FXS and typical development, even after accounting for MLU. These findings reflect both similarities and differences between individuals with DS or FXS and contribute to our understanding of the language phenotype of DS. Implications for interventions to promote language development and academic achievement are discussed.

## Introduction

Narrative language competence, the ability to generate or retell a personal or fictional story, is a fundamental aspect of spoken language ability. In addition to its importance in maintaining cohesive conversational interactions in social situations (McCabe and Bliss, 2003; Reed and Spicer, 2003), narrative competence plays a central role in school achievement (Dickinson and McCabe, 2001). Termed a “bridge to literacy,” narrative competence scaffolds the development of both reading comprehension and writing (McCabe and Peterson, 1991). The study reported here focused on evaluating the narrative competence of individuals with Down syndrome (DS), who because of several phenotypic characteristics associated with DS, lead to the hypothesis that narrative will be especially challenging for them. We examined narrative performance in individuals with DS relative to fragile X syndrome (FXS), another neurodevelopmental disorder that causes intellectual disability and delays in spoken language, so as to evaluate the syndrome specificity of our findings in DS.

Most individuals with DS have moderate to severe intellectual disability and pervasive language impairments. Across domains of language, comprehension is generally less impaired than expression, with some aspects of comprehension (e.g., vocabulary knowledge) commensurate with levels of nonverbal cognitive ability (Abbeduto et al., 2003). Expressive language, however, is impaired relative to nonverbal cognition (Chapman and Hesketh, 2000; Abbeduto et al., 2007). Social functioning is a relative strength for individuals with DS, especially in terms of their willingness to interact with a variety of social partners (Fidler et al., 2008). Further, children with DS are often regarded by others as friendly and affectionate (Gibbs and Thorpe, 1983). Despite this sociability, however, older children and adolescents with DS exhibit problems with higher-order cognitive processing of social information (Fidler, 2006; Cebula et al., 2010) and often experience difficulties navigating interpersonal interactions (Channell et al., 2015). Limited reciprocal friendships are reported during adolescence, and meaningful employment during adulthood is often not achieved (Iarocci et al., 2008).

For individuals with DS, it is likely that narrative competence will both affect and be affected by the linguistic and social cognitive impairments associated with DS. Telling a well-developed narrative requires the coordination of a complex set of skills across multiple developmental domains. For example, children must be able to integrate and organize their everyday experiences into mental representations of events. In conversation with a listener, they must be able to hold these events in mind while using spoken language to represent temporal and causal relationships in a coherent manner (Lahey and Bloom, 1994; Berman, 1995). Furthermore, narrative requires perspective taking and inferences about the mental states (e.g., emotions, plans, and goals) of story characters as well as predictions about character actions and reactions (Trabasso and Nickels, 1992). Thus, narrative language provides a window into children’s development across the cognitive, linguistic, and social pragmatic domains (Hemphill et al., 1991; Berman and Slobin, 1994; Johnels et al., 2013). The present study was designed to identify areas of relative strength and weakness in the narrative skills of individuals with DS, thereby informing treatments to enhance narrative as well as spoken language competence more broadly. Such treatments also could be useful in promoting academic and social success in this population.

We examined the narrative language samples of individuals with DS at both the macrostructural and microstructural levels of analysis. Narrative macrostructure involves evaluating the events expressed in children’s stories and the overall sequential organization of these story components (Ukrainetz et al., 2005). Narrative microstructure involves evaluating the lexical and grammatical structures that children use to convey story content (Justice et al., 2006).

### Narrative Macrostructure in DS

Only a few studies have examined macrostructural narrative skills in individuals with DS (Boudreau and Chapman, 2000; Miles and Chapman, 2002; Kay-Raining Bird et al., 2008; Finestack et al., 2012; Hogan-Brown et al., 2013), and these studies have yielded inconsistent results. These inconsistencies may reflect differences in the experimental contexts used to elicit the narratives (e.g., wordless picture books, silent films, or single or multi-scene pictures), whether picture support was available to participants during the narrative retell, and the comparison group(s) to which participants were matched. For example, when matched by nonverbal mental age, participants with DS are likely to display relative impairments in expressive language, making it important to account for expressive syntax in addition to nonverbal cognition.

Accordingly, Boudreau and Chapman (2000) evaluated event structure (defined as the mention of key plot line components) in individuals with DS who were matched to typically developing (TD) children using either nonverbal mental age, syntax comprehension, or expressive syntax [i.e., mean length of utterance (MLU)]. When asked to recall the story presented in a silent film, participants with DS conveyed more story events in their narratives than TD participants matched by MLU but not those matched by nonverbal mental age or syntax comprehension. These findings support the premise that narrative language competence in DS is dependent upon both an understanding of story content and the ability to formulate sentences to express story meaning.

Similarly, Miles and Chapman (2002), using a participant sample overlapping with that of Boudreau and Chapman (2000), showed participants a wordless picture book (*Frog, Where Are You?*) from the series written by Mercer Mayer that have been adopted for collecting narrative language samples due to their detailed illustrations, clear event structure, and character reactions. While viewing the book, the participants with DS mentioned more plot line components and search theme elements in their narratives than a TD comparison group matched by MLU, but fewer search theme elements than a TD group matched by nonverbal mental age. Thus, individuals with DS express a higher level of conceptual knowledge in their narratives than would be expected based on their expressive language levels, but not necessarily based on their nonverbal cognitive ability.

More recently, Hogan-Brown et al. (2013) analyzed narrative story structure in individuals with autism, FXS, DS, or TD, all matched on a receptive/expressive vocabulary composite. Participants were shown a storybook (*A Bed Full of Cats*, adapted into a wordless picture book) and retold the story to an examiner while viewing the pages a second time. The authors found no significant group differences in a composite score that included the number of episodes mentioned, the number of references to the story theme, and mention of a resolution. This null finding, however, is difficult to interpret because the receptive/expressive language composite matching criterion potentially conflated differences that may have varied systematically by participant group. That is, individuals with DS, FXS, and autism display unique profiles of strength and weakness in components of receptive relative to expressive language; matching based on a composite may have overshadowed some of the more subtle, yet meaningful, between-syndrome differences and made it difficult to interpret the relative role of either component in aspects of their narrative macrostructure.

Finally, Finestack et al. (2012) used the Narrative Scoring Scheme (Heilmann et al., 2010) to broadly evaluate narrative macrostructure (including the use of Introductions, Conflict/Resolution, and Cohesion of events) in verbally fluent adolescents and young adults with DS or FXS and younger TD children. Participants viewed another Mercer Mayer wordless picture book, *Frog Goes to Dinner*, and retold the story to an examiner while viewing the book pages a second time. When matched by nonverbal mental age, the participants with DS outperformed the TD participants and performed similarly to the participants with FXS; however, when matched by MLU, there was not a group difference in overall scores. These findings suggest that, despite lower levels of expressive language (i.e., MLU), exposure to a greater variety of life events (as reflected by their older chronological ages) may have helped the participants with DS or FXS convey their stories in a more sophisticated manner relative to the younger TD children. The authors noted, however, that the criterion of an MLU of at least 3.0 morphemes resulted in a restricted sample of participants with DS and may not reflect the heterogeneity observed in this population. Also, the holistic metric of narrative macrostructure, rather than one based on frequency of occurrence of specific narrative elements, may not have been nuanced enough to capture more subtle aspects of narrative that could further differentiate the participant groups.

In sum, the prior studies of narrative macrostructure in DS have utilized global approaches to evaluating the sequential organization of participant narratives. More global themes, however, are comprised of sequentially organized lower-order units called episodes, which, in turn, have their own internal organization. An alternative approach to measuring story organization could examine the mention of key elements organized *within* multiple episodes of a story. To this end, we developed an episode-based coding scheme to examine narrative macrostructure from stories produced in response to the wordless picture books *Frog Goes to Dinner* and *Frog on His Own*.

### Narrative Microstructure in DS

Some studies have taken a microstructural approach to analyzing narrative language in individuals with DS by evaluating the linguistic structures used to communicate their narratives, focusing on MLU (expressive syntax) as well as sentence complexity. For example, Hesketh and Chapman (1998) found that children with DS produced significantly fewer grammatical verbs (e.g., forms of *do*, *be*, or *have*) and main verbs per utterance relative to TD children matched by MLU. Further, participants with DS who had MLUs in excess of 3.5 words produced a significantly higher number of different main, but not grammatical, verbs. These results suggest that MLU may play a different role in verb use in DS than in typical development. However, a comparison group of individuals with intellectual disability of another origin (e.g., FXS) is needed to determine the syndrome specificity of this finding.

Evaluating other microstructural components, Chapman et al. (1998) found that children, adolescents, and young adults with DS omitted more words than TD children matched by nonverbal mental age. Most of the words omitted by participants with DS were function words that contributed to the syntactic complexity of sentences (e.g., verb forms, articles, prepositions, pronouns, adverbs, and conjunctions). More recently, however, Thordardottir et al. (2012) found that narratives produced by older children and adolescents with DS (a subset of the sample reported by Chapman et al., 1998) did not show differences in measures of sentence complexity compared to younger TD children matched by MLU. The authors did, however, note particularly wide variability in performance within the group with DS.

In all of the microstructure-focused studies to date, narratives were collected in unstructured conversations, with variability in narrative contexts across participants even within a single study. In the Hesketh and Chapman (1998) study, for example, about two-thirds of participant narratives came from talking about a favorite book or activity, whereas the remainder consisted of retellings of a wordless picture book. Such variability makes interpretation of the findings more difficult, as these types of task differences are likely to be confounded with participant group. Moreover, the skills needed for conveying a personal narrative are likely to differ from those needed to retell a fictional story, especially if the story-telling context includes picture supports in the form of illustrations from the book.

In the current study, we analyzed aspects of narrative microstructure in youth with DS in the context of narration of a wordless picture book. We focused our microstructural analysis specifically on the use of verbs, conjunctions, and adverbs because these word classes have particular relevance to the ability to communicate event sequences that tie together the story grammar elements considered in our macrostructural analyses. The existing literature also does not inform us as to whether observed patterns of narrative microstructure are specific to the language phenotype of DS or more common to intellectual disability in general, and the relative role of MLU is still unclear. Thus, in the current study, we compared narrative microstructure in youth with DS to youth with FXS as well as younger TD children of similar nonverbal cognitive ability levels and statistically evaluated the role of MLU in the analyses.

### Current Study Aims

We used both macrostructural and microstructural approaches to evaluate the narratives produced by children and adolescents with DS in response to wordless picture books. Specifically, we addressed the following research questions. (1) Is there a strength or weakness at the macrostructural level in story grammar organization in youth with DS relative to youth with FXS or TD children of similar nonverbal cognitive ability level? (2) What is the relative role of MLU (i.e., expressive syntax) in story grammar organization in youth with DS relative to the comparison groups? (3) At the microstructural level, do youth with DS differentially use grammatical word categories in their narratives relative to youth with FXS or TD children? (4) What is the relative role of MLU in the use of different word categories in youth with DS relative to the comparison groups?

These data will contribute to ongoing efforts to further characterize the DS phenotype by identifying areas of relative strength and difficulty in spoken language use. Ultimately, greater specification of narrative language development in this population should lead to the development of more effectively targeted treatments.

## Materials and Methods

### Participants

Participants were drawn from a larger study on language development in neurodevelopmental disorders and overlap with those described in previous studies (e.g., Kover et al., 2012; Finestack et al., 2013); however, all of the analyses reported in this paper have not been previously conducted or reported. In the larger study, inclusion criteria were parent report that the child used speech as a primary mode of communication, was a native English speaker, could produce at least three-word phrases in everyday speech, functioned generally at the kindergarten level or higher, and had no major uncorrected physical or sensory impairments that would interfere with the ability to perform in the project. Additionally, all participants were required to pass a hearing screening indicating a pure-tone threshold of <30 dB in at least one ear. For the present study, we also required that each participant have complete data on the Narrative Task, defined as story-relevant speech on at least 75% of the book page spreads. Seven individuals with DS did not meet this criterion due to non-compliance/lack of task completion and thus were excluded from the present study.

Participants with DS were matched to a sample of youth with FXS (*t*_(43)_ = −0.332, *p* = 0.742) and a sample of TD children (*t*_(44)_ = −0.058, *p* = 0.954) who were selected on the basis of nonverbal cognitive ability level (i.e., Leiter-R growth score values; see Table 1). The sample with FXS and the TD sample were also matched to each other on nonverbal cognitive ability level (*t*_(43)_ = 0.274, *p* = 0.785). All participants with FXS or TD who were selected into the comparison samples also met the present study’s inclusion criteria listed above. This resulted in a final sample of 23 youth with DS (10–16 years old; 13 males, 10 females), 22 youth with FXS (10–16 years old; 19 males, 3 females), and 23 TD children (3–6 years old; 14 males, 9 females).

**Table 1:** **Descriptive characteristics by participant group**.

	DS *Mean (SD) Range*	FXS *Mean (SD) Range*	TD *Mean (SD) Range*
Chronological age	12.80 (1.59)	12.33 (1.74)	4.48 (0.86)
	10.28–15.54	10.18–16.01	3.11–6.19
Leiter-R growth scores	462.09 (7.66)	462.82 (7.09)	462.22 (7.58)
	442–474	446–476	442–474
Leiter-R standard scores^a^	42.48 (7.07)	44.41 (7.87)	110.96 (15.50)
	36–65	36–65	87–159
MLU in morphemes	5.07 (2.00)	5.11 (1.42)	6.19 (1.32)
	1.40–9.17	2.83–7.37	4.07–8.83
TROG-2 raw scores	24.00 (9.34)	40.91 (17.62)	25.50 (13.41)
	5–42	11–65	7–64

*^a^DS: n = 7, FXS: n = 5, TD: n = 0 scored at the floor standard score of 36*.

For participants with DS, we relied on parent report or, when available, a copy of a karyotype or physician report of a diagnosis of DS. Documentation was not available for five participants, but the remainder was documented as Trisomy 21. For participants with FXS, we required written documentation of an FMR1 full mutation based on molecular genetic testing. For the TD participants, we required that they had no diagnosis of a developmental disability and were not receiving special education services. Additionally, none of the TD participants were receiving speech/language therapy. Participants were recruited through a university registry, postings on websites and listservs, newspaper ads, and in the case of the TD children, local preschools. Approval for human subjects research was granted by the affiliated universities’ institutional review boards, and written consent was obtained from parents/guardians of all participants.

### Measures

#### Narrative Task

Participants were shown one of two wordless picture books, *Frog Goes to Dinner* (Mayer, 1974) or *Frog on His Own* (Mayer, 1973), and then told the story to an examiner while viewing the book a second time. The book version was counterbalanced across participants in the larger study. For the initial viewing of the book, each participant was told to look at the pictures so s/he could see what happened in the story, and the examiner turned the pages of the book so that each page spread was viewed for approximately 10 s. For the retell, the participant was instructed to tell the examiner everything about the story, page by page. Examiner prompts were scripted to minimize examiner scaffolding of the narrative retell. The examiner controlled the page turns and waited 5–7 s after each participant response prior to turning to the next page. Participants’ narratives were audio-recorded for later transcription. The examiner said “next page” at each page turn so that transcribers were aware of the location in the book during transcription.

#### Transcription of Participant Narratives

Trained personnel transcribed audio files of participants’ narratives verbatim using Systematic Analysis of Language Transcription (SALT; Miller and Iglesias, 2006) software. For each narrative language sample, a primary transcriber completed a first draft and then a second transcriber listened to the language sample, checked the transcription draft, and provided feedback to the primary transcriber, who was responsible for finalizing the transcript. Transcribers were highly trained according to the procedures described by Abbeduto et al. (1995). Transcribers segmented participants’ speech into communication units (C-units), defined as an independent clause and its modifiers, which can include dependent clauses (Loban, 1976). Segmentation into C-units provides a more accurate measure of language ability than segmentation into utterances for children beyond the developmental age of 3 years (Abbeduto et al., 1995). As reported by Kover et al. (2012) and Finestack et al. (2013), approximately 20% of narrative transcripts from each participant group was checked for inter-transcriber agreement, averaging 94% for TD participants, 90% for participants with FXS, and 86% for participants with DS [averaging over C-unit segmentation, intelligibility, mazes, overlaps, pauses, abandonment, word identification, number of morphemes and words, and ending punctuation (e.g., question intonation)].

#### Coding Story Grammar Use

Following transcription, trained study personnel coded narrative transcripts for the presence of pre-defined story grammar elements within episodes of *Frog Goes to Dinner* or *Frog on His Own*, using the coding scheme described below. We applied the story grammar paradigm set forth by Stein and Glenn (1975) to structure our coding scheme. For each book, we identified one setting at the beginning of the story, which established the location and/or timeframe of the entire story. Then, we divided each book into five episodes. For each episode, we identified the following six story grammar elements: an *Initiating Event* that triggers the episode, a character’s *Internal Response* to the initiating event, the character’s *Plan* or goal in response to the initiating event, the character’s *Attempt* to put the plan into action, an *Outcome* or consequence resulting from the character’s action, and a character’s physical or psychological *Reaction* to the outcome (see Table 2). To guide our interpretation of the story elements in the episodes, we tallied the components of each story mentioned by a group of older TD children whose narratives were considered a “gold standard” child sample. This approach, along with reviewing the story scripts provided by SALT, provided the framework for developing the final episodic structure of each story.

**Table 2:** **Story grammar elements**.

	Definition	Example^a^
Initiating event	Event or problem that kicks off the episode	Frog jumps in (or is in) the saxophone
Internal response	Reference to character’s psychological state in response to the initiating event	Musician wonders (or doesn’t know) what happened
Plan	Reference to character’s intent to act upon or resolve the problem caused by the initiating event	Musician wants to figure out why his saxophone won’t play
Attempt	Character action directed at carrying out the plan	Musician tips over saxophone to look inside
Outcome	Consequence of the attempt (may not resolve the problem)	Frog lands/is on the musician’s face
Reaction	Character reaction to the outcome (emotions or actions)	Musician falls into the drum/The drummer is angry

*^a^Example from Episode 2 of Frog Goes to Dinner*.

Using this coding scheme, trained personnel reviewed each participant’s transcript and independently identified the story grammar elements that were included in the narrative for each episode. Coders were blind to the participant’s diagnostic group. Coders used a copy of the book as a reference, which, along with the page numbers provided on the transcripts, allowed them to confirm the pages that were being referenced by the participant during any part of the narrative. Coders awarded credit for any given element if the child provided enough story content on the appropriate page of the book to allow the coder to identify which element s/he was referencing. Although abandoned C-units were excluded, C-units with unintelligible segments were considered if there was enough information in the C-unit to determine its meaning. A child did not need to mention one element in order to receive credit for the next. Credit was only awarded once for each story element within an episode. If a child used more than one C-unit to relay a story element, those C-units could be considered together, but s/he still only received one point for that element. Additionally, in some episodes there were numerous ways in which a child could receive credit for a given story element. If the child correctly referenced more than one example, again s/he only received one point for the element. Thus, one initial setting plus six elements within each of five episodes resulted in a maximum story grammar score of 31 points for either book.

Approximately 20% of the narrative transcripts from each book were coded independently by a second coder to assess reliability. Point-by-point inter-coder agreement averaged 93% (range = 85–100%) for *Frog Goes to Dinner* and 96% (range = 90–100%) for *Frog on His Own*. See Table 3 for inter-coder agreement by story grammar element type.

**Table 3:** **Average point-by-point inter-coder agreement for story grammar elements**.

	*Frog Goes to Dinner (%)*	*Frog on His Own (%)*	Total (%)	
Initiating events	97.78	95.00	96.47
Attempts	91.11	100.00	95.29
Outcomes	91.11	92.50	91.76
Reactions	91.11	97.50	94.12

#### Coding Grammatical Word Category Use

We coded transcripts from participants’ narratives for the use of main verbs, adverbs, and conjunctions. Semantic context was taken into consideration such that a participant did not receive credit for a word used in a nonsensical or non story-related semantic context. However, a participant received credit for a semantically appropriate use of a word even if the C-unit was not syntactically correct (e.g., “frog jump inside” would receive credit for the verb *jump*). Although abandoned C-units were excluded, C-units with unintelligible segments were included if there was enough information in the C-unit to determine whether a word was used in an appropriate semantic context. Scores were calculated as the proportion of C-units containing each word category. Approximately 20% of the narrative transcripts from each book were coded independently by a second coder to assess reliability. Point-by-point inter-coder agreement averaged 96% (range = 83–100%) for *Frog Goes to Dinner* and 97% (range = 86–100%) for *Frog on His Own*.

#### Mean Length of Utterance (MLU)

We used SALT software to compute participants’ MLU (i.e., mean length of C-units) in morphemes from each participant’s narrative transcript. Only complete and fully intelligible C-units were included in this computation. Thus, abandoned C-units and C-units with unintelligible segments were excluded because it is not possible to determine how many morphemes were produced within an unintelligible segment.

#### Leiter International Performance Scale-Revised (Leiter-R; Roid and Miller, 1997)

The Leiter-R is a standardized measure of nonverbal cognition that is nonverbal in administration and in participant response method. We used growth scores from the Brief IQ screener as a metric of nonverbal cognitive ability level for participant matching. Growth scores are scaled corrections of raw scores that take into account item difficulty but reflect absolute ability level rather than an age-based norm. This is of particular relevance for individuals with intellectual disability who may perform at the floor level of standard scores (Hessl et al., 2009). For ease of interpretation, however, we also report standard scores in the participant descriptives. The Leiter-R Brief IQ screener correlates with the Wechsler Intelligence Scale for Children, Third Edition (Wechsler, 1991) at *r* = 0.85, and reported reliability of the Leiter-R is *r* = 0.88. The Leiter-R is normed for ages 2–21 years.

#### Test for Reception of Grammar, Second Edition (TROG-2; Bishop, 2003)

The TROG-2 is a standardized measure of receptive syntax. Participants were instructed to point to pictures that best represented phrases or sentences spoken by an examiner. Due to extensive floor effects on standard scores, we report raw scores in the description of the sample characteristics of our participant groups. Reported internal consistency reliability of the TROG-2 is 0.88.

## Results

### Sample Descriptive Characteristics

See Table 1 for descriptive characteristics of our sample by participant group. There was no significant difference between the participants with DS and those with FXS in terms of chronological age (*t*_(43)_ = 0.940, *p* = 0.353) or nonverbal IQ (*t*_(43)_ = −0.866, *p* = 0.391). There also was no significant difference in MLU between the participants with DS and those with FXS, *t*_(43)_ = −0.080, *p* = 0.937. The participants with DS, however, displayed significantly lower MLUs than the TD participants, *t*_(44)_ = −2.236, *p* = 0.030. In terms of receptive syntax (i.e., TROG-2 raw scores), there was not a statistically significant difference between the groups with DS and FXS, *t*_(43)_ = −0.437, *p* = 0.664. The participants with DS, again however, exhibited significantly lower receptive syntax abilities than the TD participants, *t*_(44)_ = −4.132, *p* < 0.001.

### Macrostructural Analyses

#### Story Grammar Organization

We used a nested regression model to examine the relation of diagnostic group to overall story grammar organization scores and to evaluate the contribution of MLU. Using DS as the reference group, we used dummy codes so that the binary variable “TD” represented the TD-DS comparison and the binary variable “FXS” represented the FXS-DS comparison. These diagnostic group variables were included in Step 1 of the regression model, and MLU was included in Step 2. An examination of residuals indicated no major violations of the assumptions of linear regression. The resulting model was significant, *F*_(3,67)_ = 34.552, *p* < 0.001, *R*^2^ = 0.618, with the full model accounting for 62% of the variance in story grammar scores.

In the first step, diagnostic group accounted for a significant amount of the variance in the model, *F*_(2,67)_ = 3.219, *p* = 0.046, *R*^2^ = 0.090. An examination of the standardized coefficients indicated that the TD-DS contrast was significant (*β* = 0.331, *p* = 0.018), but the FXS-DS contrast was not significant (*β* = 0.077, *p* = 0.572); thus, the TD group had significantly higher story grammar scores than the group with DS, but there was no significant difference in scores between the groups with FXS and DS.

The addition of MLU in the second step also accounted for significant unique variance to the model, *F-change*_(1,64)_ = 88.547, *p* < 0.001, *R*^2^ change = 0.528. An examination of the standardized coefficients revealed that with MLU added into the model (*β* = 0.765, *p* < 0.001), the diagnostic group contrasts were no longer significant (TD-DS *β* = 0.087, *p* = 0.350; FXS-DS *β* = 0.068, *p* = 0.445). Thus, after accounting for MLU, the difference in story grammar scores between the TD group and the group with DS was no longer significant. See Table 4 for story grammar scores by participant group.

**Table 4:** **Primary analyses: narrative scores by participant group**.

	DS *Mean (SD) Range*	FXS *Mean (SD) Range*	TD *Mean (SD) Range*
**Macrostructural variables^a^**
*Story grammar organization*	6.52 (5.70)	7.36 (3.91)	10.09 (5.10)
	0–19	1–14	0–17
**Microstructural variables^b^**
*Verb use*	0.50 (0.28)	0.61 (0.22)	0.74 (0.18)
	0.00–1.00	0.24−0.96	0.39−0.98
*Adverb use*	0.19 (0.18)	0.19 (0.16)	0.35 (0.23)
	0.00−0.76	0.00−0.51	0.04−0.78
*Conjunction use*	0.03 (0.06)	0.03 (0.05)	0.07 (0.10)
	0.00−0.18	0.00−0.15	0.00−0.41

*^a^Number of episodic elements expressed (maximum = 31). ^b^Proportion of C-units including the word category*.

#### Inclusion of Story Grammar Elements

To further analyze story grammar organization in the participants’ narratives, we explored whether there was a difference among the participant groups in their inclusion of the different types of story grammar elements. For these analyses, we examined the number of episodes (0–5) in which participants included each type of story grammar element; however, because internal responses and plans were rarely mentioned by any of our participants, we did not analyze them further. For the remaining elements, we used nonparametric analyses because of the limited range of scores and the violation of the assumption of normality in their distributions. See Table 5 for median scores by element type for each group.

**Table 5:** **Exploratory analyses: use of story grammar element type by participant group**.

	DS *Median (Range)*	FXS *Median (Range)*	TD *Median (Range)*
Setting^a^	34.8% used	52.2% used	45.5% used
Initiating Event^b^	3 (0–5)	3 (0–5)	4 (0–5)
Internal Response^b^	0 (0–1)	0 (0–1)	0 (0–2)
Plan^b^	0 (0–1)	0 (0–0)	0 (0–1)
Attempt^b^	1 (0–4)	1 (0–3)	2 (0–4)
Outcome^b^	1 (0–4)	1 (0–3)	1 (0–4)
Reaction^b^	1 (0–5)	2 (0–4)	2 (0–5)

*^a^Scored as present/absent for entire story. ^b^Scored as present/absent for each of the five episodes*.

#### Settings

We used a 2 × 3 chi-square analysis to determine if there was a difference among participant groups in whether or not they mentioned a setting in their narratives. This analysis was not statistically significant, *X*^2^_(2)_ = 1.434, *p* = 0.488, indicating no differences among groups in their use of a setting.

#### Initiating Events

A Kruskal-Wallis test, used to explore whether there was a difference among participant groups in how many Initiating Events they mentioned, was not statistically significant, *H*_(2)_ = 4.149, *p* = 0.126, indicating no between group differences.

#### Attempts

A Kruskal-Wallis test, used to explore whether there was a difference among participant groups in how many Attempts they mentioned, was marginally significant, *H*_(2)_ = 5.525, *p* = 0.063. Given the exploratory nature of these analyses, we conducted one-tailed Mann-Whitney *post hoc* analyses to explore whether there were fewer Attempts used by the group with DS compared to the TD group or the group with FXS. Results indicated that the group with DS used significantly fewer Attempts than the TD group (*U* = 179.000, *Z* = −1.922, *p* = 0.028) but not the group with FXS (*U* = 232.500, *Z* = −0.489, *p* = 0.315).

#### Outcomes

A Kruskal-Wallis test, used to explore whether there was a difference among groups in how many Outcomes they mentioned, was not statistically significant, *H*_(2)_ = 3.129, *p* = 0.209, indicating no group differences.

#### Reactions

A Kruskal-Wallis test, used to explore whether there was a difference among groups in the number of Reactions mentioned, was not statistically significant, *H*_(2)_ = 4.318, *p* = 0.115, again indicating no group differences.

### Microstructural Analyses

#### Use of Grammatical Word Categories

To determine whether there were group differences in the use of the different grammatical word categories (proportionate to the total number of C-units produced), we created three nested regression models: one predicting verb use, one predicting adverb use, and one predicting conjunction use. For each model, with DS as the reference group, the dummy-coded binary variables of diagnostic group (“TD” and “FXS”) were the independent variables. Again, we included MLU in the second step of the model to evaluate its contribution. An examination of residuals indicated no major violations of the assumptions of linear regression. See Table 4 for proportions of word category use by participant group.

#### Verb Use

The model predicting verb use was significant, *F*_(3,67)_ = 45.601, *p* < 0.001, *R*^2^ = 0.681, accounting for 68% of the total variance. In the first step, group contributed significant variance, *F*_(2,67)_ = 6.008, *p* = 0.004, *R*^2^ = 0.158. The standardized coefficients revealed that the TD-DS contrast was significant (*β* = 0.457, *p* = 0.001), but the FXS-DS contrast was not (*β* = 0.216, *p* = 0.104), indicating a significantly lower rate of verb use by the group with DS relative only to the TD group. The inclusion of MLU in the second step accounted for a significant amount of additional variance in the model, *F-change*_(1,64)_ = 105.122, *p* < 0.001, *R*^2^ change = 0.524. With MLU in the model (*β* = 0.762, *p* < 0.001), both diagnostic group contrasts became significant (TD-DS *β* = 0.214, *p* = 0.014; FXS-DS *β* = 0.207, *p* = 0.013). Thus, after accounting for MLU, the rate of verb use was significantly lower in the group with DS relative to both the TD group and the group with FXS.

#### Adverb Use

The model predicting adverb use was significant, *F*_(3,67)_ = 25.619, *p* < 0.001, *R*^2^ = 0.546, accounting for 55% of the variance. In the first step, group accounted for a significant portion of the variance, *F*_(2,67)_ = 5.159, *p* = 0.008, *R*^2^ = 0.137. The standardized coefficients revealed that the TD-DS contrast was significant (*β* = 0.359, *p* = 0.009), but the FXS-DS contrast was not (*β* = −0.021, *p* = 0.873), indicating a significantly lower rate of adverb use by the group with DS relative only to the TD group. The inclusion of MLU in the second step accounted for a significant amount of additional variance in the model, *F-change*_(1,64)_ = 57.560, *p* < 0.001, *R*^2^ change = 0.409. The standardized coefficients revealed that, with MLU in the model (*β* = 0.673, *p* < 0.001), neither diagnostic group contrast remained significant (TD-DS *β* = 0.145, *p* = 0.156; FXS-DS *β* = −0.029, *p* = 0.765).

#### Conjunction Use

The model predicting conjunction use was significant, *F*_(3,67)_ = 15.385, *p* < 0.001, *R*^2^ = 0.419, accounting for 42% of the total variance. In the first step, however, group did not contribute significant variance, *F*_(2,67)_ = 1.537, *p* = 0.223, *R*^2^ = 0.045, indicating that neither the TD-DS (*β* = 0.203, *p* = 0.151) nor the FXS-DS (*β* = −0.018, *p* = 0.895) group differences in conjunction use was significant. The inclusion of MLU in the second step accounted for a significant amount of variance in the model, *F-change*_(1,64)_ = 41.179, *p* < 0.001, *R*^2^ change = 0.374. With MLU in the model (*β* = 0.644, *p* < 0.001), the group contrast variables were still not significant (TD-DS *β* = −0.002, *p* = 0.985; FXS-DS *β* = −0.236, *p* = 0.814). However, it should be noted that across participant groups, conjunction use was very low (Table 4).

## Discussion

The present study was designed to examine the macrostructrual and microstructural aspects of narratives produced by children and adolescents with DS. Narrative is a foundational skill for learning to use language to interact with others (McCabe and Bliss, 2003; Reed and Spicer, 2003) and is a scaffold for the acquisition of literacy-related skills and academic achievement (Dickinson and McCabe, 2001). Thus, our study was designed to inform work on the behavioral phenotype of DS and provide insights into potential targets for interventions that could have positive consequences for the daily functioning of individuals with DS.

Unlike previous studies in this area, we focused on the mastery of the internal organization of the episodes that serve as the building blocks of a story. In general, individuals with DS expressed fewer of the elements of episodic structure than did younger TD children of similar nonverbal cognitive levels, suggesting that this aspect of narrative macrostructure is especially impaired in DS. We also found, however, that the difference in expression of episodic elements between youth with DS and TD children was eliminated once the difference in MLU, or syntactic competence, was controlled. This finding suggests that individuals with DS have acquired the conceptual knowledge needed to express the key story elements (at least to the level expected for their nonverbal cognitive ability), but that their limited expressive syntactic abilities limit their ability to put that knowledge into words during the course of telling a story. This conclusion is consistent with the findings of previous studies suggesting that individuals with DS can sometimes express conceptually more mature narratives than TD peers when expressive abilities are equated through participant selection and or statistical control (Boudreau and Chapman, 2000; Miles and Chapman, 2002). In the current study, however, this result occurred despite the fact that, in our coding scheme, a participant was awarded credit for a story element even if it was communicated over two or three short utterances rather than in one utterance. It would appear, then, that there is a need for interventions targeting narrative language competence in DS and that such interventions should provide models of, and practice with, a range of linguistic options for expressing episodic structure.

In typical development, children begin to use individual story grammar elements in their narratives during the early preschool years. Most 3-year-olds only describe isolated pictures, mentioning only the most salient aspects of the story in a fragmented manner. Older preschool-aged children begin to communicate event sequences and connect the initiating event with an outcome in the story, sometimes also mentioning character actions that mediate the initiating event and the outcome/consequence. By around 5 or 6 years of age, children can formulate temporally organized event sequences and are able to communicate overarching story themes. Story grammar organization continues to progress during the school-age years as children develop a stronger cognitive framework for event sequencing and for talking about character goals and plans (Karmiloff-Smith, 1981; Bamberg, 1987; Bamberg and Marchman, 1990; Reilly, 1992; Berman and Slobin, 1994). The findings from our study suggest that in DS, story grammar organization may develop closely with and rely critically on expressive grammar.

Our exploratory analyses revealed that the difficulty observed in story grammar in the participants with DS may be centered on the expression of Attempts, which are in some ways the core of an episode, representing the actions taken by story characters to deal with the problem or dilemma that launched the episode. Because Attempts are actions that are motivated by a character’s goals and other internal states, the results suggest that individuals with DS have difficulty talking about others’ perspectives and intentions, an idea compatible with the growing body of literature on the social behavioral phenotype of DS (Fidler et al., 2005; Fidler, 2006; Cebula et al., 2010; Hahn et al., 2013). The lack of communication of character actions could also stem from a difficulty in verb production that has been observed in DS in other language sampling contexts (Hesketh and Chapman, 1998; Michael et al., 2012), as verbs are necessary to communicate character actions. Although verbs are also important for communicating other story grammar elements (e.g., plans or reactions), action verbs in particular are needed to encode and express character attempts/actions. Regardless of explanation, it would appear that interventions targeting narrative language competence in DS should include an emphasis on the expression of Attempts. This could include focusing on skills such as event sequencing or perspective taking, and narrative storytelling provides an optimal context for scaffolding such skills.

We also found that the narratives of individuals with DS did not differ from those of individuals with FXS, at least in terms of the aspects of narrative macrostructure we examined. This finding suggests that the impairments in narrative macrostructure we examined are not specific to DS, but may be associated with intellectual disability more generally. Beyond their intellectual disability, individuals with DS and FXS share a delay in spoken language. Although there are marked differences in the specific language profiles observed across the two disorders (e.g., Abbeduto et al., 2003), the findings from this study suggest a shared deficit in the use of story grammar.

In terms of narrative microstructure, we examined the expression of three major syntactic categories of words—verbs, adverbs, and conjunctions—all of which are critical for the expression of episodic structure as well as other dimensions of narrative macrostructure. More specifically, verbs allow children to talk about overt character actions (e.g., *run*, *catch*, *jump*) as well as character psychological states, including their plans and goals (e.g., *want*, *hope*) or emotional reactions (e.g., *laugh*, *cry*). Conjunctions aid event sequencing by linking events in temporal (e.g., “He took a drink *after* he jumped in the glass”) and causal (e.g., “He fell over *because* the bee stung his tongue”) relationships. Adverbs (i.e., words that describe where, when, how, etc.), much like conjunctions, also play an important role in accurately describing events in the context of place and time (e.g., *over there*, *next*) as well as allowing the child to use evaluative devices that enable the speaker to make comments about the story to the listener (e.g., “He *really* didn’t like it”, “The frog *always* got in trouble”).

Individuals with DS were less likely to use adverbs and verbs in their stories than were their TD cognitively matched peers. After controlling for variation in MLU, the group difference in the rate of adverb use was no longer significant. Controlling for MLU, however, did not eliminate the DS-TD difference in rate of verb use. Further, when controlling for MLU, the difference between the groups with DS and FXS also became significant, with the participants with DS showing a lower rate of verb use. This suggests that beyond their general impairment in expressive grammar, individuals with DS exhibit a specific deficit in verb production that may contribute to their unique behavioral phenotype rather than being general to intellectual disability. Indeed, problems in verb mastery have been documented in DS in other studies as well (Hesketh and Chapman, 1998; Chapman, 2003), and our findings extend this to the context of narrative storytelling from a book and by documenting an impairment not shared by those with FXS. Given the action-oriented nature of Attempts, it is likely that limitations in expressive mastery of verbs may be contributing to the macrostructure impairments displayed by individuals with DS. Thus, interventions designed to improve narrative competence in this population should also pay particular attention to modeling new action verbs in the service of expressing narrative content. This would provide individuals with DS with the linguistic tools needed to express the key story elements, particularly Attempts, in their narratives, thus improving their narrative language at the macrostructural level as well.

The finding regarding the relative role of MLU to group differences in verb use further suggests that for individuals with DS, other mechanisms may be driving their development of verb use. For example, it could be that the specific weakness in phonological memory that is characteristic of many individuals with DS plays a role in their ability to learn verbs, which often appear in the middle of a spoken sentence and thus are more difficult to encode (see Naigles et al., 1995; but see Miolo et al., 2005). Another possibility is that a difficulty in abstract learning may be driving this deficit. That is, the abstract nature of verbs requires children to learn the word they hear by mapping it to a transitory action they observe, a much less concrete task than mapping a label (i.e., a noun) to an object that remains in front of them. Before any conclusions can be drawn, however, more research is needed to identify such potential predictors of verb learning in DS. Because verbs are so integral to the ability to effectively communicate events and personal experiences to others, this is an area worthy of further investigation.

Beyond contributing to a better understanding of narrative abilities in DS, our study also highlights a new approach to measuring both macrostructural and microstructural narrative abilities of individuals with intellectual disability that can be used in phenotypic research as well as for measuring change over time (e.g., in response to a language intervention). With the recent focus on developing outcome measures that are appropriate for individuals with intellectual disability across a wide age range (e.g., Berry-Kravis et al., 2013b), the Narrative Task (Abbeduto et al., 1995) employed in this study has received much attention. This task provides a naturalistic context for measuring spoken language (i.e., storytelling from a picture book), while also providing a standardized context for administration. Because it is not subject to the same floor effects and compliance problems that occur with many standardized assessments of spoken language in these populations, it is an ideal candidate for use as an outcome measure. In fact, several research studies have documented its utility as such, showing that the expressive language measures derived from this task (e.g., MLU, vocabulary diversity, talkativeness, etc.) can discriminate typical from atypical populations, distinguish different genetic syndromes associated with intellectual disability, and show excellent test-retest reliability (Abbeduto et al., 1995; Finestack and Abbeduto, 2010; Kover et al., 2012; Berry-Kravis et al., 2013a). The current study goes beyond the standard expressive language measures that can be derived from the Narrative Task and demonstrates its ability to detect individual differences, as well as differences among typical and atypical samples, in aspects of narrative language competence at the macrostructural and microstructural levels of analysis.

There are, however, limitations to the present study. First, the small sample sizes of the participant groups and the exploratory nature of the analyses by story grammar element type suggest that the results should be interpreted with caution and are in need of replication. Future research should also test the generalizability of the findings to the broader population with DS (e.g., other age ranges and ability levels). Although the age range of participants in the present study is ideal for examining narrative language competence, in particular, the results may be less applicable to individuals with DS who are younger and/or less verbal than the present sample. Furthermore, we did not screen for comorbid diagnoses such as autism spectrum disorder that could also affect the generalizability of the results. Finally, participant groups were not matched on sex, another important factor to consider in future work.

In sum, this study extended prior work on narrative language in DS by taking a new approach to measuring their macrostructural and microstructural narrative abilities and by adding a same-age comparison group of individuals with intellectual disability of another origin (i.e., FXS). Importantly, this study provides a new method for researchers to capture individual differences across a wide range of ages and ability levels of individuals with intellectual disability, including those with DS. By no means, however, did this study capture all of the macrostructural and microstructural narrative language abilities of individuals with DS. For example, the use of evaluative devices that engage the listener, such as sound effects or character dialog, would provide additional insight into their story-telling abilities. Furthermore, researchers should consider the use of inferential language (e.g., mental state language; predictions; causal referencing) in the narratives produced by individuals with DS, as this would provide more information regarding their perspective taking and abstract reasoning skills. Finally, researchers should also consider using videos to capture non-linguistic communication acts (e.g., gestures or facial expressions) that children with DS may be using to communicate their stories to a listener. Ultimately, data on narrative provide an exciting new avenue for intervention, both in terms of informing clinicians where to target during intervention to promote spoken language development and in equipping them with a way to capture change in the use of those skills over time within the naturalistic context of shared book reading.

## Author Contributions

MMC and ASM conceptualized the study’s design, developed the coding schemes reported in the manuscript, and had primary roles in data analysis and interpretation and in drafting the manuscript. LMB helped develop the coding scheme, assisted with data analysis, and helped draft the manuscript. LA participated in the study’s conception and design, assisted in data interpretation and drafting the manuscript, and provided mentorship throughout the project. All authors have read and approved the final version of this manuscript.

## Conflict of Interest Statement

Leonard Abbeduto has received financial support to develop and implement outcome measures for clinical trials from F. Hoffman-LaRoche Ltd., Roche TCRC Inc., and Neuren Pharmaceuticals Ltd. The other authors declare that the research was conducted in the absence of any commercial or financial relationships that could be construed as a potential conflict of interest.

## References

[B1] Abbeduto L. Benson G. Short K. Dolish J. (1995). Effects of sampling context on the expressive language of children and adolescents with mental retardation. Ment. Retard. 33, 279–288. 7476250

[B2] Abbeduto L. Murphy M. M. Cawthon S. W. Richmond E. K. Weissman M. D. Karadottir S.. (2003). Receptive language skills of adolescents and young adults with down or fragile X syndrome. Am. J. Ment. Retard. 108, 149–160. 10.1352/0895-8017(2003)108<0149:rlsoaa>2.0.co;2 12691594

[B3] Abbeduto L. Warren S. F. Conners F. A. (2007). Language development in down syndrome: from the prelinguistic period to the acquisition of literacy. Ment. Retard. Dev. Disabil. Res. Rev. 13, 247–261. 10.1002/mrdd.20158 17910087

[B4] Bamberg M. (1987). The Acquisition of Narratives. Berlin: Mouton de Gruyter.

[B5] Bamberg M. Marchman V. (1990). What holds a narrative together? The linguistic encoding of episode boundaries. Pap. Pragmat. 4, 58–121. 10.1075/iprapip.4.1-2.02bam

[B6] Berman R. A. (1995). Narrative competence and storytelling performance: how children tell stories in different contexts. J. Narrat. Life Hist. 5, 285–313. 10.1075/jnlh.5.4.01nar

[B7] Berman R. A. Slobin D. I. (1994). Relating Events in Narrative: A Crosslinguistic Developmental Study. Hillsdale, NJ: Lawrence Erlbaum.

[B8] Berry-Kravis E. Doll E. Sterling A. Kover S. T. Schroeder S. M. Mathur S.. (2013a). Development of an expressive language sampling procedure in fragile X syndrome: a pilot study. J. Dev. Behav. Pediatr. 34, 245–251. 10.1097/dbp.0b013e31828742fc 23669871 PMC3654391

[B9] Berry-Kravis E. Hessl D. Abbeduto L. Reiss A. L. Beckel-Mitchener A. Urv T. K. (2013b). Outcome measures for clinical trials in fragile X syndrome. J. Dev. Behav. Pediatr. 34, 508–522. 10.1097/dbp.0b013e31829d1f20 24042082 PMC3784007

[B10] Bishop D. V. M. (2003). Test for Reception of Grammar, Version 2. London: Psychological Corporation.

[B11] Boudreau D. M. Chapman R. S. (2000). The relationship between event representation and linguistic skill in narratives of children and adolescents with down syndrome. J. Speech Lang. Hear. Res. 43, 1146–1159. 10.1044/jslhr.4305.1146 11063236

[B12] Cebula K. R. Moore D. G. Wishart J. G. (2010). Social cognition in children with down’s syndrome: challenges to research and theory building. J. Intellect. Disabil. Res. 52, 113–134. 10.1111/j.1365-2788.2009.01215.x 19874447

[B13] Channell M. M. Phillips B. A. Loveall S. J. Conners F. A. Bussanich P. M. Klinger L. G. (2015). Patterns of autism spectrum symptomatology in children and adolescents with down syndrome without comorbid autism spectrum disorder. J. Neurodev. Disord. 7:5. 10.1186/1866-1955-7-5 25657824 PMC4318440

[B14] Chapman R. S. (2003). Language and communication in individuals with down syndrome. Int. Rev. Res. Ment. Retard. 27, 1–34. 10.1016/s0074-7750(03)27001-4

[B15] Chapman R. S. Hesketh L. J. (2000). Behavioral phenotype of individuals with down syndrome. Ment. Retard. Dev. Disabil. Ret. Rev. 6, 84–95. 10.1002/1098-2779(2000)6:2<84::aid-mrdd2>3.0.co;2-p 10899801

[B16] Chapman R. S. Seung H. Schwartz S. E. Kay-Raining Bird E. (1998). Language skills of children and adolescents with down syndrome: II. Production deficits. J. Speech Lang. Hear. Res. 41, 861–873. 10.1044/jslhr.4104.861 9712133

[B17] Dickinson D. K. McCabe A. (2001). Bringing it all together: the multiple origins, skills and environmental supports of early literacy. Learn. Disabil. Res. Pract. 16, 186–202. 10.1111/0938-8982.00019

[B18] Fidler D. J. (2006). The emergence of a syndrome-specific personality profile in young children with down syndrome. Downs Syndr. Res. Prac. 10, 53–60. 10.3104/reprints.305 16869362

[B19] Fidler D. J. Most D. E. Booth-LaForce C. Kelly J. F. (2008). Emerging social strengths in young children with down syndrome. Infants Young Child. 21, 207–220. 10.1097/01.iyc.0000324550.39446.1f

[B20] Fidler D. J. Philofsky A. Hepburn S. L. Rogers S. J. Abbeduto L. (2005). Nonverbal requesting and problem-solving by toddlers with down syndrome. Am. J. Ment. Retard. 110, 312–322. 10.1352/0895-8017(2005)110[312:nrapbt]2.0.co;2 15941367 PMC4512657

[B21] Finestack L. H. Abbeduto L. (2010). Expressive language profiles of verbally expressive adolescents and young adults with down syndrome or fragile X syndrome. J. Speech Lang. Hear. Res. 53, 1334–1348. 10.1044/1092-4388(2010/09-0125) 20643789 PMC2948067

[B22] Finestack L. H. Palmer M. Abbeduto L. (2012). Macrostructural narrative language of adolescents and young adults with down syndrome or fragile X syndrome. Am. J. Speech Lang. Pathol. 21, 29–46. 10.1044/1058-0360(2011/10-0095) 22049405 PMC3273569

[B23] Finestack L. H. Sterling A. M. Abbeduto L. (2013). Discriminating down syndrome and fragile X syndrome based on language ability. J. Child Lang. 40, 244–265. 10.1017/s0305000912000207 23217297 PMC3532435

[B24] Gibbs M. V. Thorpe J. G. (1983). Personality stereotype of noninstitutionalized down syndrome children. Am. J. Ment. Defic. 87, 601–605. 10.1016/s0022-3476(76)80398-8 6223525

[B25] Hahn L. J. Fidler D. J. Hepburn S. L. Rogers S. J. (2013). Early intersubjective skills and the understanding of intentionality in young children with down syndrome. Res. Dev. Disabil. 34, 4455–4465. 10.1016/j.ridd.2013.09.027 24112996 PMC3882942

[B26] Heilmann J. Nockerts A. Miller J. F. (2010). Language sampling: does the length of the transcript matter? Lang. Speech Hear. Serv. Sch. 41, 393–404. 10.1044/0161-1461(2009/09-0023) 20601531

[B27] Hemphill L. Picardi N. Tager-Flusberg H. (1991). Narrative as an index of communicative competence in mildly mentally retarded children. Appl. Psycholinguist. 12, 263–279. 10.1017/s014271640000922x

[B28] Hesketh L. J. Chapman R. S. (1998). Verb use by individuals with down syndrome. Am. J. Ment. Retard. 103, 288–304. 10.1352/0895-8017(1998)103<0288:vubiwd>2.0.co;2 9833659

[B29] Hessl D. Nguyen D. V. Green C. Chavez A. Tassone F. Hagerman R. J.. (2009). A solution to limitations of cognitive testing in children with intellectual disabilities: the case of fragile X syndrome. J. Neurodev. Disord. 1, 33–45. 10.1007/s11689-008-9001-8 19865612 PMC2768415

[B30] Hogan-Brown A. L. Losh M. Martin G. E. Mueffelmann D. J. (2013). An investigation of narrative ability in boys with autism and fragile X syndrome. Am. J. Intellect. Dev. Disabil. 118, 77–94. 10.1352/1944-7558-118.2.77 23464607 PMC3602926

[B31] Iarocci G. Yager J. Rombough A. McLaughlin J. (2008). The development of social competence among persons with down syndrome: from survival to social inclusion. Int. Rev. Res. Ment. Retard. 35, 87–119. 10.1016/s0074-7750(07)35003-9

[B32] Johnels J. A. Hagberg B. Gillberg C. Miniscalco C. (2013). Narrative retelling in children with neurodevelopmental disorders: is there a role for nonverbal temporal-sequencing skills? Scand. J. Psychol. 54, 376–385. 10.1111/sjop.12067 23855443

[B33] Justice L. M. Bowles R. P. Kaderavek J. N. Ukrainetz T. A. Eisenberg S. L. Gillam R. B. (2006). The index of narrative microstructure: a clinical tool for analyzing school-age children’s narrative performances. Am. J. Speech Lang. Pathol. 15, 177–191. 10.1044/1058-0360(2006/017) 16782689

[B34] Karmiloff-Smith A. (1981). “The grammatical marking of thematic structure in the development of language production,” in The Child’s Construction of Language, ed Deutsch W. (London: Academic Press), 121–147.

[B35] Kay-Raining Bird E. Cleave P. L. White D. Pike H. Helmkay A. (2008). Written and oral narratives of children and adolescents with down syndrome. J. Speech Lang. Hear. Res. 51, 436–450. 10.1044/1092-4388(2008/032) 18367688

[B36] Kover S. T. McDuffie A. Abbeduto L. Brown W. T. (2012). Effects of sampling context on spontaneous expressive language in males with fragile X syndrome or down syndrome. J. Speech Lang. Hear. Res. 55, 1022–1038. 10.1044/1092-4388(2011/11-0075) 22232386 PMC3337358

[B37] Lahey M. Bloom L. (1994). “Variability and language learning disabilities,” in Language Learning Disabilities in School-Age Children and Adolescents: Some Principles and Applications, eds Wallach G. P. Butler K. G. (Merrill: University of California), 354–372.

[B38] Loban W. (1976). Language Development: Kindergarten through Grade Twelve. Urbana, IL: National Council of Teachers of English.

[B39] Mayer M. (1973). Frog on His Own. New York: Dial Books for Young Readers.

[B40] Mayer M. (1974). Frog Goes to Dinner. New York: Dial Books for Young Readers.

[B55] McCabe A. Peterson C. (1991). Developing Narrative Structure. Hillsdale, NJ: Lawrence Erlbaum.

[B41] McCabe A. Bliss L. S. (2003). Patterns of Narrative Discourse: A Multicultural, Life Span Approach. Boston: Pearson.

[B42] Michael S. E. Ratner N. B. Newman R. (2012). Verb comprehension and use in children and adults with down syndrome. J. Speech Lang. Hear. Res. 55, 1736–1749. 10.1044/1092-4388(2012/11-0050) 22992705

[B43] Miles S. Chapman R. S. (2002). Narrative content as described by individuals with down syndrome and typically developing children. J. Speech Lang. Hear. Res. 45, 175–189. 10.1044/1092-4388(2002/013) 14748647

[B44] Miller J. Iglesias A. (2006). Systematic Analysis of Language Transcripts (SALT Version 9) [Computer Software]. Madison, WI: Language Analysis Lab, University of Wisconsin-Madison.

[B45] Miolo G. Chapman R. S. Sindberg H. A. (2005). Sentence comprehension in adolescents with down syndrome and typically developing children: role of sentence voice, visual context and auditory-verbal short-term memory. J. Speech Lang. Hear. Res. 48, 172–188. 10.1044/1092-4388(2005/013) 15938064

[B46] Naigles L. G. Fowler A. Helm A. (1995). “Syntactic bootstrapping from start to finish with special reference to down syndrome,” in Beyond Names for Things: Young Children’s Acquisition of Verbs, eds Tomasello M. Merriman W. E. (Hillsdale, NJ: Lawrence Erlbaum), 299–330.

[B47] Reed V. A. Spicer L. (2003). The relative importance of selected communication skills for adolescents’ interactions with their high school teachers: high school teachers’ opinions. Lang. Speech Hear. Serv. Sch. 34, 343–357. 10.1044/0161-1461(2003/028) 27764462

[B48] Reilly J. S. (1992). How to tell a good story: the intersection of language and affect in children’s narrative. J. Narrat. Life Hist. 2, 355–377. 10.1075/jnlh.2.4.04how

[B49] Roid G. Miller L. (1997). Leiter International Performance Scale-Revised. Wood Dale, IL: Stoelting.

[B50] Stein N. L. Glenn C. G. (1975). “An analysis of story comprehension in elementary school children: a test of a schema,” in Multidisciplinary Approaches to Discourse Rrocessing, ed. Freedle R. (Norwood, NJ: Ablex), 53–120.

[B51] Thordardottir E. T. Chapman R. S. Wagner L. (2012). Complex sentence production by adolescents with down syndrome. Appl. Psycholinguist. 23, 163–183. 10.1017/s0142716402002011

[B52] Trabasso T. Nickels M. (1992). The development of goal plans of action in the narration of a picture story. Discourse Processes 15, 249–275. 10.1080/01638539209544812

[B53] Ukrainetz T. A. Justice L. M. Kaderavek J. N. Eisenberg S. L. Gillam R. B. Harm H. M. (2005). The development of expressive elaboration in fictional narratives. J. Speech Lang. Hear. Res. 48, 1363–1377. 10.1044/1092-4388(2005/095) 16478377

[B54] Wechsler D. (1991). WISC-III: Wechsler Intelligence Scale for Children. 3rd Edn. San Antonio: Psychological Corporation.

